# Breast cancer screening

**DOI:** 10.1590/0100-3984.2023.56.4e1-en

**Published:** 2023

**Authors:** Luciano Fernandes Chala, Linei Augusta Brolini Delle Urban

**Affiliations:** 1 Coordinator of the National Mammography Commission, Colégio Brasileiro de Radiologia e Diagnóstico por Imagem (CBR), São Paulo, SP, Brazil; 2 Member of the National Mammography Commission, Colégio Brasileiro de Radiologia e Diagnóstico por Imagem (CBR), São Paulo, SP, Brazil

Breast cancer is one of the most commonly diagnosed tumors worldwide^(1)^. In recent decades, advances in disease screening and adjuvant therapies have resulted in a reduction in the mortality associated with breast cancer. The goal of breast cancer screening is to detect the disease early, before it becomes symptomatic. Early detection reduces the need for more extensive clinical or surgical approaches, as well as increasing the likelihood that the treatment will be efficacious, because the disease might not yet have progressed. However, the paradigms of breast cancer screening have changed from a one-size-fits-all approach, based mainly on the age of the woman, to a more individualized approach, which incorporates breast density and the risk for future breast cancer development, as well as new imaging technologies.

In this issue of Radiologia Brasileira, the *Colégio Brasileiro de Radiologia e Diagnóstico por Imagem* (CBR, Brazilian College of Radiology and Diagnostic Imaging), the *Sociedade Brasileira de Mastologia* (SBM, Brazilian Society of Mastology), and the *Federação Brasileira das Associações de Ginecologia e Obstetrícia* (FEBRASGO, Brazilian Federation of Gynecological and Obstetrical Associations) publish their recommendations for breast cancer screening in Brazil^(2)^, with relevant changes in relation to the last update, which was published in 2017^(3)^. Among those changes is a specific section created for women with dense breasts and a recommendation that supplementary biennial screening with magnetic resonance imaging (MRI) be considered for women with extremely dense breasts, given that, in recent years, studies such as the Dense Trial^(4)^ have demonstrated the importance of supplementary screening in such women. There are also relevant changes in the recommendations for women with a personal history of lobular neoplasia, atypical ductal hyperplasia, or breast cancer. In the case of women carrying a mutation in the BRCA1 gene, the recommendation was changed to screening by mammography starting at age 35, with screening by MRI alone between the ages of 25 and 35, thus increasing the benefits of early screening and reducing risk. It was reaffirmed the importance of annual screening by mammography for women at average risk starting from the age of 40, because of the higher prevalence of breast cancer in this age group in lowand middle-income countries, as well as a trend toward an increase in the number of cases of breast cancer diagnosed in women under 50 years of age, led to a recent discussion and review of the recommendations of the United States Preventive Services Task Force^(5)^.

The objective of the CBR/SBM/FEBRASGO update was to incorporate the best recent scientific evidence in order to further reduce the mortality associated with breast cancer, by promoting its detection as early as possible, while taking into account the risks of breast cancer screening.

One important aspect of modern breast cancer screening is its increased complexity. That requires greater material and human resources, which are not widely available in Brazil. Many facilities in the country will not be able to offer supplemental screening, especially with MRI. Therefore, the search for alternatives that can reduce costs, such as abbreviated MRI of the breasts^(6)^, should be encouraged. Tomosynthesis, understood as an evolved form of mammography with significant benefits, such as an increase in the detection rate and fewer false positives^(7)^, is not widely available in Brazil. Access to percutaneous biopsy, especially that guided by MRI, is also still quite limited in Brazil, despite its importance in the investigation of lesions that can be identified only by that method. Finally, the quality of the examinations still needs to be improved, mainly by encouraging participation in programs for quality control or auditing.

In conclusion, although the CBR/SBM/FEBRASGO update of the recommendations for breast cancer screening represents progress, there are obstacles to its full implementation in a country such as Brazil. To provide women in Brazil with access to the best possible screening for breast cancer, those obstacles must be overcome, and they can be overcome through the unified efforts of Brazilian society as a whole.
